# Clinical assessment and three-dimensional movement analysis: An integrated approach for upper limb evaluation in children with unilateral cerebral palsy

**DOI:** 10.1371/journal.pone.0180196

**Published:** 2017-07-03

**Authors:** Lisa Mailleux, Ellen Jaspers, Els Ortibus, Cristina Simon-Martinez, Kaat Desloovere, Guy Molenaers, Katrijn Klingels, Hilde Feys

**Affiliations:** 1 KU Leuven–University of Leuven, Department of Rehabilitation Sciences, Leuven, Belgium; 2 Neural Control of Movement Lab, Department of Health Sciences and Technology, ETH Zurich, Zurich, Switzerland; 3 KU Leuven–University of Leuven, Department of Development and Regeneration, Leuven, Belgium; 4 University Hospitals Leuven, Clinical Motion Analysis Laboratory, Leuven, Belgium; 5 University Hospitals Leuven, Department of Orthopaedic Medicine, Leuven, Belgium; 6 UHasselt–Hasselt University, BIOMED, Rehabilitation Research Center (REVAL), Diepenbeek, Belgium; Semmelweis Egyetem, HUNGARY

## Abstract

**Introduction:**

The clinical application of upper limb (UL) three-dimensional movement analysis (3DMA) in children with unilateral cerebral palsy (uCP) remains challenging, despite its benefits compared to conventional clinical scales. Moreover, knowledge on UL movement pathology and how this relates to clinical parameters remains scarce. Therefore, we investigated UL kinematics across different manual ability classification system (MACS) levels and explored the relation between clinical and kinematic parameters in children with uCP.

**Patients and methods:**

Fifty children (MACS: I = 15, II = 26, III = 9) underwent an UL evaluation of sensorimotor impairments (grip force, muscle strength, muscle tone, two-point discrimination, stereognosis), bimanual performance (Assisting Hand Assessment, AHA), unimanual capacity (Melbourne Assessment 2, MA2) and UL-3DMA during hand-to-head, hand-to-mouth and reach-to-grasp tasks. Global parameters (Arm Profile Score (APS), duration, (timing of) maximum velocity, trajectory straightness) and joint specific parameters (angles at task endpoint, ROM and Arm Variable Scores (AVS)) were extracted. The APS and AVS refer respectively to the total amount of movement pathology and movement deviations of wrist, elbow, shoulder, scapula and trunk.

**Results:**

Longer movement durations and increased APS were found with higher MACS-levels (p<0.001). Increased APS was also associated with more severe sensorimotor impairments (r = -0.30-(-0.73)) and with lower AHA and MA2-scores (r = -0.50-(-0.86)). For the joint specific parameters, stronger movement deviations distally were significantly associated with increased muscle weakness (r = -0.32-(-0.74)) and muscle tone (r = 0.33-(-0.61)); proximal movement deviations correlated only with muscle weakness (r = -0.35–0.59). Regression analysis exposed grip force as the most important predictor for the variability in APS (p<0.002).

**Conclusion:**

We found increased movement pathology with increasing MACS-levels and demonstrated the adverse impact of especially muscle weakness. The lower correlations suggest that 3DMA provides additional information regarding UL motor function, particularly for the proximal joints. Integrating both methods seems clinically meaningful to obtain a comprehensive representation of all aspects of a child’s UL functioning.

## Introduction

Unlike gait analysis, the clinical application of upper limb (UL) three-dimensional movement analysis (3DMA) remains challenging in children with unilateral cerebral palsy (uCP). In children with uCP, UL function has thus far been extensively studied using reliable and valid clinical scales for bimanual performance or unimanual capacity such as the Assisting Hand Assessment [1] or the Melbourne Assessment [2], respectively. Notwithstanding their clinical and scientific value, these clinical scales lack quantitative data as their scores are based on visual observations and they only provide limited information on selective anatomical motions and movement patterns at the individual joint levels. In contrast, a more detailed and objective description can be obtained by means of 3DMA with which the amount of movement pathology can be captured [3].

Studies using UL 3DMA have reported more wrist flexion, and more elbow pronation and flexion in children with uCP when reaching for a vertically oriented cylinder, resulting in aberrant shoulder kinematics and increased trunk movements compared to typically developing children [3–7]. Also during more functional tasks, such as hand-to-mouth and hand-to-head, deviant UL kinematics have been reported [3–6,8,9]. Furthermore, children with uCP have longer movement durations, less straight hand trajectories and lower maximum velocities when executing UL tasks compared to their typically developing peers [3,5,6,8,10]. Lastly, within the group of children with uCP, lower manual abilities have also been related to longer movement durations [6,10], less straight hand trajectories and more severe UL movement pathology [10,11] such as increased elbow pronation and trunk flexion [6]. Whilst these studies offer first insights into the relation between manual abilities and movement pathology, results are based on small sample sizes and incomplete UL kinematic descriptions [6,10,11]. Additionally, a detailed mapping of UL kinematics according to manual ability in children with uCP is still lacking.

Thus far, only one study investigated the relation between UL kinematics and motor impairments. Jaspers et al. [12] found increasing UL movement pathology with decreasing functional level and increasing muscle weakness and tone. However, these authors only explored correlations with total movement pathology, and used a smaller sample size. Hence, knowledge of the impact of muscle weakness or muscle tone on movement deviations at the individual joint level is still very limited. Moreover, the relation between UL kinematics and measures of bimanual performance or unimanual capacity has not yet been investigated. Conventional clinical scales mostly focus on distal UL motor function, and a further investigation of their relation with UL movement pathology will undoubtedly increase our understanding of the role of proximal versus distal movement pathology with respect to UL functioning. In the long run, these insights will allow creating individualized treatment plans and thus aid in the further optimization of UL therapy.

The first aim of this study was therefore to map UL movement pathology in children with uCP according to Manual Ability Classification System (MACS) [13] levels. Secondly, we aimed to investigate the role of UL sensorimotor impairments (muscle strength, muscle tone, sensory impairments) in UL kinematics and to explore the relation between bimanual performance, unimanual capacity and UL movement pathology.

## Materials and methods

### Participants

Children with a spastic type of uCP were recruited via the CP-care program of the University Hospitals Leuven (Belgium). Children were prospectively enrolled if they were aged between 5 and 15 years, able to comprehend test instructions and could at least actively grasp an object. Exclusion criteria were botulinum toxin-A injections in the 6 months prior to testing or a history of UL surgery. The protocol was approved by the Ethical Committee of the University Hospitals Leuven (S50480, S55555), and parental written informed consent was obtained for all children prior to participation. Children age 12 years or older were additionally asked for their assent prior to participation.

### Procedure

All children underwent a comprehensive UL evaluation including a conventional clinical assessment of sensorimotor impairments, an evaluation of bimanual performance and unimanual capacity, and an UL 3DMA at the Clinical Motion Analysis Laboratory of the University Hospitals Leuven. Children were assessed by three well-trained physiotherapists who were routinely involved in the clinical evaluation of children with CP.

### Clinical assessment

Descriptive characteristics such as age, gender, impaired side and MACS level were collected. Sensorimotor impairments were evaluated according to a standardized and reliable protocol described by Klingels et al. [14]. Muscle tone was assessed with the Modified Ashworth Scale [15] in six muscle groups at the level of the shoulder (adductors, internal rotators), elbow (flexors, pronators), wrist and hand (wrist and finger flexors) (total score; 0–24). Muscle strength was measured using the Medical Research Council rating for four muscle groups at the level of the shoulder (abductors), elbow (extensors and supinators) and wrist (extensors) (total score; 0–20). Grip force was evaluated with the Jamar dynamometer (Lafayette Instrument Company, Lafayette, IN) and the ratio of the mean of three maximum contractions of the impaired versus the less-impaired hand was used for further analysis. Sensory assessments included two-point discrimination (TPD) and stereognosis [14]. TPD was evaluated as the minimal distance at which one or two points were correctly distinguished using an aesthesiometer at the distal phalanx of the index finger. Stereognosis was assessed through tactile identification of six objects.

To evaluate bimanual performance, the Assisting Hand Assessment (AHA) [1] was used. The AHA assesses the spontaneous use of the impaired hand in bimanual activities during a semi-structured play session which is video-recorded. Afterwards, 22 items are scored and converted to 0–100 logit-based AHA units. The Melbourne Assessment 2: a test of unilateral upper limb function (MA2) [2] was used to assess unimanual capacity. This criterion-referenced test evaluates four elements of UL movement quality: range of motion (ROM), accuracy, dexterity and fluency. It contains 14 unimanual tasks which are video-recorded for subsequent scoring. Raw scores are converted to a percentage score for each of the four sub-scales.

### Three-dimensional movement analysis

UL kinematic analysis was performed following the protocol described by Jaspers et al. [5,12,16]. Seventeen reflective markers were attached to the trunk (n = 3), acromion (n = 3), humerus (n = 4), forearm (n = 4) and hand (n = 3). The starting position was upright sitting with 90° of hip and knee flexion, which was ensured with a custom-made chair with adjustable foot and back support. All recordings were done with 12 to 15 infrared Vicon-cameras sampling at 100 Hz. Static calibration trials were first performed to identify the anatomical landmarks as described by Wu et al. [17]. Next, children were asked to perform the following dynamic trials: (1) hand-to-head (HTH), (2) hand-to-mouth (HTM) and (3) reach-to-grasp a vertically oriented cylinder (RGV). The cylinder was placed at shoulder height and arm length distance. All tasks were executed with the impaired UL at self-selected speed. Each task was repeated four times within one single recording, and two successful recordings were collected per task. This resulted in eight movement repetitions per task. After data collection, start (i.e. hand on ipsilateral knee) and end positions (i.e. point of task achievement, PTA) of the movement repetitions were identified using Nexus software (Oxford Metrics, Oxford, UK). For each dynamic trial, two movement repetitions were selected for further analyses, depending on the child’s task compliance and marker visibility (i.e. movement repetitions with marker occlusions >20% of the movement duration were excluded). All UL kinematics were calculated in MATLAB using U.L.E.M.A. (v1.1.9, available for download at https://github.com/u0078867/ulema-ul-analyzer).

For every task, global parameters (Arm Profile Scores (APS) and spatiotemporal parameters) and joint specific parameters (Arm Variable Scores (AVS), endpoint angles and active ROM)) were extracted. The *APS and AVS* were determined as described in Jaspers et al. [12]. The AVS was calculated for 13 joint angles as the root mean square error (RMSE) between the point-by-point comparison of each joint angle of the child with uCP and that same joint angle of a reference database (N = 20 typically developing children, age 5–15 years). The RMSE-average of all 13 joint angles equals the APS. The APS is thus considered an index of overall severity of UL movement pathology, the 13 AVS represent the deviating scores for the wrist (flexion/extension, ulnar/radial deviation), elbow (flexion/extension, pronation/supination), shoulder (elevation plane, elevation, rotation), scapula (anterior/posterior tilting, medial/lateral rotation, pro/retraction) and trunk (flexion/extension, lateral bending, axial rotation). *Spatiotemporal parameters* included movement duration, timing of maximum velocity, maximum velocity and trajectory straightness (calculated as the ratio of the actual length of the travelled hand path and the direct linear distance between start and endpoint). Finally, *joint angles at point of task achievement* (PTA) and *total active ROM* during task execution were extracted from the joint angular time-series for each specific joint movement.

### Statistical analysis

Descriptive statistics were used to document demographic, clinical and UL kinematic characteristics. First, differences in UL movement pathology across MACS levels were investigated using a Kruskal-Wallis test with post-hoc Mann-Whitney U tests. To account for dependencies between the joint specific parameters (AVS, angles at PTA and ROM), the sequentially rejective Holm-Bonferroni method [18] was applied for every joint angle for the Kruskall-Wallis tests. The post-hoc significance level was also corrected using the Holm-Bonferroni method. Secondly, correlation coefficients were calculated between clinical and kinematic parameters using pearson (r_p_) or biserial (r_b_) correlation coefficients, depending on the type of data. Correlation coefficients <0.30 were considered as little or no correlation, 0.30 to 0.50 low, 0.50 to 0.70 moderate, >0.70 high and 0.90 to 1.00 very high [19]. The significance levels were corrected to account for the dependencies between the joint specific parameters (AVS, angles at PTA and ROM) using the Holm-Bonferroni method. Finally, a stepwise multiple regression analysis was used to identify which variables explained the variability in APS for all three tasks. Variables entered in the regression model were age, MACS level, grip force, muscle strength and muscle tone. The level of significance was set at p<0.05, with the multiple level of sequentially rejective significance levels set to three combinations (α_1_<0.0167, α_2_<0.025, α_3_<0.05). Statistical procedures were carried out with SAS Enterprise Guide 7.1 (SAS Institute Inc., Cary, NC, USA).

## Results

### Participants

Fifty children with uCP were enrolled in this study (mean age 10 years, 5 months ± 2 years, 8 months; 32 boys; 28 left side impaired). Fifteen children were categorized as MACS I, 26 as MACS II and 9 as MACS III. Age did not differ statistically between the three MACS groups (p = 0.28). Clinical characteristics according to MACS levels are presented in supporting information (S1 Table). Clinical assessments and 3DMA were performed on the same day, except for eight children (time gap < 8 months). These eight children were included in the analyses, as no significant time effects for these assessments have been reported for a period of up to one year [20]. Six children had missing data for the AHA and MA2.

### Movement pathology across MACS levels

#### Global parameters

**Fig 1:**
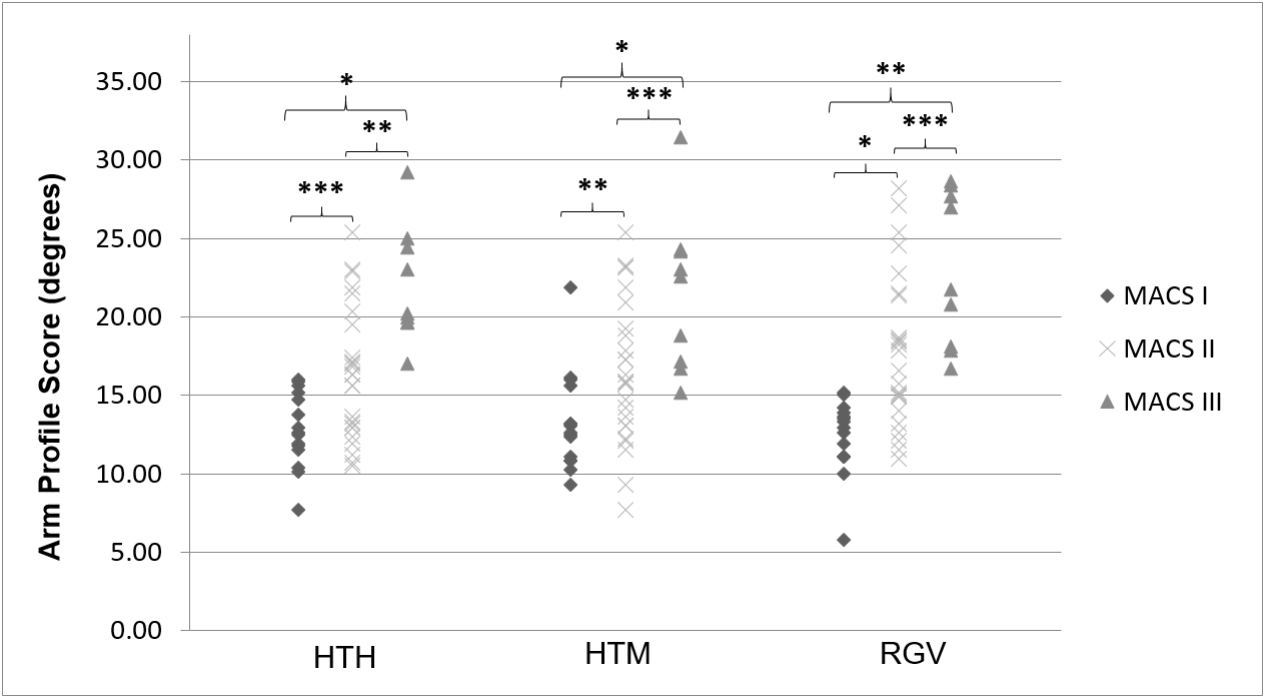
Statistical comparison of the Arm Profile Score between MACS I, II and III for all tasks. HTH, hand-to-head; HTM, hand-to-mouth; RGV, reach-to-grasp vertically, MACS; Manual Ability Classification System; Holm-Bonferroni sequential significance levels * p<0.0167; ** p<0.025; *** p<0.05.

**Fig 2:**
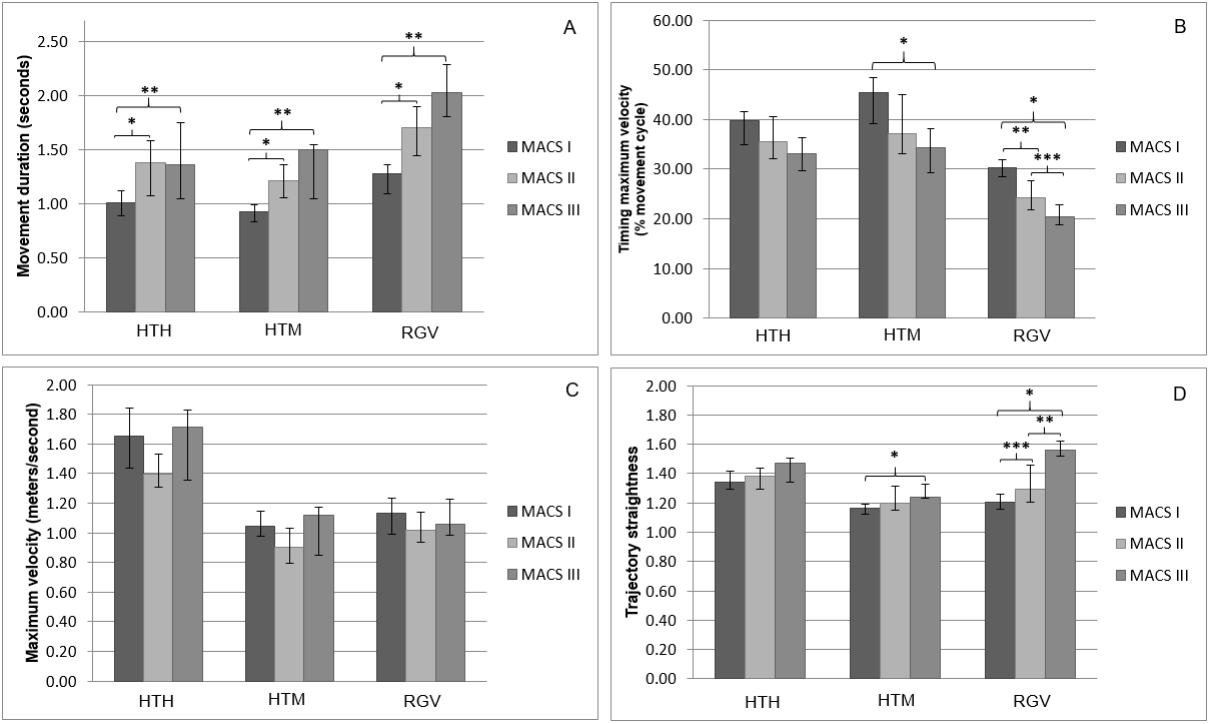
Statistical comparison of spatiotemporal parameters between MACS I, II and III for all tasks. 2A, movement duration; 2B, timing of maximum velocity; 2C, maximum velocity; 2D, trajectory straightness; HTH, hand-to-head; HTM, hand-to-mouth; RGV, reach-to-grasp vertically; MACS, Manual Ability Classification System; Holm-Bonferroni sequential significance levels * p<0.0167; ** p<0.025; *** p<0.05.

Higher APS and longer movement durations were found with increasing MACS levels for all three tasks (Figs 1 and 2; H = 13.06–23.39, p<0.002). Post-hoc comparisons showed significant differences between all MACS levels for the APS (U = 0–105, p<0.03) and between MACS I and II and I and III for movement duration (U = 14–77, p<0.02). During HTM and RGV, children with higher MACS levels also reached their maximum velocity earlier and showed less straight hand trajectories (Fig 2; H = 7.56–18.04, p<0.02). For HTM, post-hoc tests showed significant differences between MACS I and III (U = 23 and 16 respectively, p<0.02), while for RGV differences between all MACS levels were significant (U = 3–98, p<0.02). Maximum velocity was not significantly different between the different MACS levels for any of the tasks (Fig 2; p>0.05).

#### Joint specific parameters

Wrist flexion/extension (AVS, angle at PTA), elbow pro/supination (AVS, angle at PTA, ROM) and trunk flexion/extension (ROM) differed significantly between MACS levels for all three tasks, with more deviating values with increasing MACS levels (Tables 1–3; H = 6.77–24.63, p<0.03). A significantly larger ROM of wrist flexion was also noted for children with higher MACS levels during HTM and RGV (Tables 2 and 3, H = 8.66 and 9.18 respectively, p = 0.01). Also task specific differences were found, i.e. children with higher MACS levels used more shoulder elevation at PTA during HTM (Table 2, H = 7.01, p = 0.01) and less elbow extension (AVS, angle at PTA, ROM) during RGV (Table 3, H = 6.83–22.61, p<0.03). RGV also resulted in higher AVS for shoulder elevation, trunk lateral bending and trunk rotation in children with higher MACS levels (Table 3, H = 8.48–11.06, p<0.01). Post-hoc comparisons are shown in Tables 1–3, whereby the majority of the differences were found between MACS I and III. Waveform kinematics (mean curves) of the individual joints for all three groups are provided as online supplementary material (S1–S3 Figs).

**Table 1:** Statistical comparison of the joint specific parameters (Me, IQR) between MACS I, II and III during hand-to-head.

		MACS l (n = 15)	MACS ll (n = 26)	MACS lll (n = 9)	p-value
**WRIST flexion/extension**	AVS	**11 (8.7–14.8)**	**18.8 (14.4–32.8)**	**45.2 (41.9–50.6)**	<0.0001^*^^,^^**a**^^**,**^^**b**^^**,**^^**c**^
	PTA	**22.7 (14.1–32.2)**	**37.3 (16.0–54.3)**	**62.8 (58.0–65.4)**	0.0003^*^^,^^**b**^^**,**^^**c**^
	ROM	44.8 (30–48.4)	40.4 (29.9–51.5)	45.4 (36.2–66.2)	0.67
**WRIST ulnar/radial deviation**	AVS	14.2 (8.2–21)	10.1 (6–19.7)	15.8 (10.5–20.3)	0.21
	PTA	-15.8 (-37.8–(-5.4))	-10.6 (-17.1–0.6)	-1.3 (-29.2–7.6)	0.17
	ROM	31.5 (18.4–36.4)	21.5 (15.0–26.6)	16.9 (16.2–19.3)	0.03
**ELBOW pro/supination**	AVS	**17.1 (9.6–21.4)**	**17.3 (11.4–40.3)**	**27.5 (22.9–50.5)**	0.02^*^^,^^**b**^
	PTA	**78.7 (62.6–85.4)**	**90.0 (72.5–119.8)**	**100.1 (85.5–123.7)**	0.03^*^^,^^**d**^
	ROM	**75.7 (64–89)**	**58.6 (46.3–71.8)**	**48.3 (39.2–72.3)**	0.009^*^^,^^**a**^^**,**^^**b**^
**ELBOW flexion/extension**	AVS	9.4 (7.1–14.7)	12.2 (10.2–19.9)	13.8 (7.7–19.4)	0.17
	PTA	104.6 (98.2–114.2)	111.8 (105.3–114.6)	105.2 (103.4–109.7)	0.25
	ROM	59.8 (54.6–69.3)	59.2 (49.3–65.8)	49.6 (41.4–64.5)	0.43
**SHOULDER elevation plane**	AVS	16.8 (10.5–22.5)	14.2 (9–23.1)	18.8 (14.5–23.9)	0.65
	PTA	56.1 (43.6–65.8)	57.9 (46.8–66.0)	54.9 (49.2–62.5)	0.96
	ROM	24 (13.3–34.3)	25.1 (19.5–31.5)	26.8 (24.6–27.4)	0.58
**SHOULDER elevation**	AVS	8.6 (6–9.7)	10.5 (7.1–15.3)	14.8 (9.3–16.7)	0.13
	PTA	-99.6 (-105.3 -(-96.8))	-97.5 (-102–(-92.6))	-107 (-110.8–(-93.7))	0.14
	ROM	79.7 (72.8–86.4)	74.4 (63.5–82.3)	84.4 (59–87.4)	0.34
**SHOULDER rotation**	AVS	14.4 (8.9–19.2)	14.5 (10.4–20.5)	18.0 (8.7–24.1)	0.86
	PTA	-56.1 (-66.9–(-50.4))	-65.4 (-74.8–(-55.2))	-65.4 (-78.9–(-55.4))	0.22
	ROM	37.9 (27.5–49.1)	38.9 (23.3–49.5)	32.0 (21.2–58.5)	0.83
**SCAPULA pro/retraction**	AVS	9.6 (3.8–13.7)	11.7 (8.2–15.6)	13.8 (11.0–23.9)	0.10
	PTA	23.3 (19.8–40.9)	30.7 (26–44.5)	39.5 (29.9–55)	0.06
	ROM	13.2 (11–17.7)	14.2 (10.7–22.6)	12.9 (10.6–18.4)	0.96
**SCAPULA medial/lateral rotation**	AVS	9.3 (6.5–12.8)	8.0 (5.8–11.2)	11.6 (6.5–12.6)	0.61
	PTA	-38.3 (-51.4–(-30.1))	-35.3 (-39.9–(-25.9))	-34.4 (-42.8–(-33.3))	0.35
	ROM	46.0 (36.9–54.3)	42.9 (32.8–46.5)	43.9 (41–56.4)	0.32
**SCAPULA anterior/posterior tilting**	AVS	8.2 (4.6–14.1)	7.41 (5.27–12.75)	6.82 (4.31–7.66)	0.50
	PTA	8 (-7.2–14.4)	1.36 (-2.42–8.08)	6.27 (1.35–10.34)	0.52
	ROM	20.1 (14.6–29.2)	17.86 (12.94–25.38)	25.15 (13.54–28.06)	0.59
**TRUNK flexion/extension**	AVS	3.1 (2.9–6.3)	4.5 (3–10)	4.8 (3.7–5.3)	0.71
	PTA	-6.9 (-11.5–(-1.3))	-8.0 (-14.5–(-4.1))	-8.4 (-11.5–(-5.4))	0.51
	ROM	**5.7 (3.8–7.3)**	**8.2 (5.4–10.9)**	**9.5 (8.3–13.7)**	0.01^*^^,^^**b**^
**TRUNK lateral bending**	AVS	3.2 (2.1–4.8)	3.3 (2.2–6.4)	5.2 (4.2–5.7)	0.22
	PTA	-9.4 (-11.9–(-7.9))	-8.0 (-11.5–(-5))	-9.9 (-13.1–(-6.7))	0.37
	ROM	8.6 (6.5–10.3)	9.5 (6.6–11.2)	13.5 (9.4–15)	0.21
**TRUNK rotation**	AVS	2.6 (1.5–5.8)	4.6 (3.1–6.6)	6.0 (3.8–8.5)	0.07
	PTA	-3.6 (-7.3–(-0.7))	3.3 (-2.9–5.5)	0.9 (-1.9–6.5)	0.03
	ROM	6 (5.1–8.8)	6.2 (3.9–8.4)	8.2 (5.8–12)	0.23

Me, median; IQR, interquartile ranges; MACS, Manual Ability Classification System; AVS, arm variable score; PTA, point of task achievement; ROM, range of motion

*, significant based on Holm-Bonferroni multiple level of sequentially rejective significance levels of three combinations; significant post-hoc differences

^a^, between MACS I and II

^b^, between MACS I and III

^c^, between II and III

^d^, no significant post-hoc differences.

**Table 2:** Statistical comparison of the joint specific parameters (Me, IQR) between MACS I, II and III during hand-to-mouth.

		MACS l (n = 15)	MACS ll (n = 26)	MACS lll (n = 9)	p-value
**WRIST flexion/extension**	AVS	**11 (8.2–17.4)**	**23.2 (11.5–40.3)**	**62.1 (34.6–66.3)**	0.0001^*^^,^^**a**^^**,**^^**b**^^**,**^^**c**^
	PTA	**1 (-9.5–8.5)**	**21 (-0.4–47.7)**	**74.7 (1.5–77.4)**	0.01^*^^,^^**d**^
	ROM	**23.7 (19–30.4)**	**36.8 (23.6–46)**	**46.5 (32.7–61.1)**	0.01^*^^,^^**b**^
**WRIST ulnar/radial deviation**	AVS	18.2 (8.4–22.7)	11.0 (7.9–19)	9.3 (7.1–17.7)	0.34
	PTA	-1.7 (-29.5–10.3)	-11.0 (-17.6–6.4)	1.8 (-6.7–8.1)	0.51
	ROM	19.5 (12.7–28.3)	20.5 (14.5–29.1)	15.5 (9.1–21.6)	0.26
**ELBOW pro/supination**	AVS	**13.6 (9.7–16.5)**	**20.9 (15.3–28.5)**	**27.8 (15.4–33.8)**	0.02^*^^,^^**a**^
	PTA	**71.2 (62.1–83.3)**	**97.2 (81.4–113.8)**	**109.6 (90.9–121)**	0.003^*^^,^^**a**^^**,**^^**b**^
	ROM	**75.4 (61.4–96.7)**	**53.4 (45.4–69.6)**	**50.1 (39.4–60.5)**	0.002^*^^,^^**a**^^**,**^^**b**^
**ELBOW flexion/extension**	AVS	7.9 (7.1–11.9)	9.3 (6.6–13)	14.5 (8.5–18.3)	0.25
	PTA	**132.3 (129–136.6)**	**135.1 (129.5–139.5)**	**139.7 (137.7–143.1)**	0.02^*^^,^^**b**^
	ROM	81.4 (77.1–88.6)	78.2 (72.9–83.3)	82.1 (70.4–83.3)	0.62
**SHOULDER elevation plane**	AVS	19.0 (13.4–24.7)	15 (11.2–27.6)	20.2 (8.6–30.6)	0.86
	PTA	81.6 (72.3–95.1)	84.8 (69.7–95.2)	77.9 (59.2–87.9)	0.59
	ROM	38.4 (25.0–51)	34.8 (20.2–50.6)	32.4 (28.1–47.1)	0.71
**SHOULDER elevation**	AVS	9.4 (8.2–11.2)	10.5 (5.6–14)	13.8 (7.1–18.8)	0.73
	PTA	-45.5 (-56.6–(-43.3))	-51.7 (-68.1–(-49.1))	-63.8 (-76.7–(-60.3))	0.03
	ROM	**25.6 (21.6–35.1)**	**31.3 (24–41.1)**	**42.7 (37.6–49.2)**	0.01^*^^,^^**b**^
**SHOULDER rotation**	AVS	17.9 (9.8–22.3)	18.3 (9.3–21.1)	20 (12.3–25.1)	0.86
	PTA	-52.6 (-61.8–(-43.7))	-69.9 (-76.5–(-55.4))	-68.5 (-82.8–(-54.5))	0.03
	ROM	30.4 (14.6–40.4)	30 (21.7–35)	34.5 (29.1–44.9)	0.58
**SCAPULA pro/retraction**	AVS	8.6 (4.4–10.1)	10.3 (4.9–14.9)	9.6 (6–14)	0.68
	PTA	42.5 (35.9–47.8)	43.7 (38.7–52.7)	47.1 (42.6–49.8)	0.42
	ROM	8.8 (7–10.7)	8.2 (6.6–12)	10.9 (8.3–11.6)	0.58
**SCAPULA medial/lateral rotation**	AVS	8.9 (4.4–11.6)	7.6 (4.9–10.1)	9.2 (6.8–12.3)	0.52
	PTA	-13.1 (-24.2–(-5.7))	-11.8 (-18.5–(-6.7))	-14.2 (-23.1–(-7.1))	0.89
	ROM	19.5 (11.8–23.3)	16.4 (13.6–25.6)	24.6 (22.7–28.3)	0.02
**SCAPULA anterior/posterior tilting**	AVS	6.2 (4.2–10.1)	6 (2.6–10.3)	4 (3.7–4.9)	0.18
	PTA	-6.8 (-12.1–(-0.2))	-5.1 (-8.1–(-2.5))	-6.7 (-9.5–(-5.6))	0.77
	ROM	7.5 (5.5–9.9)	6.9 (4.6–11.8)	6.4 (5.1–9.4)	0.90
**TRUNK flexion/extension**	AVS	3.8 (1.5–4.7)	3.1 (2–4.6)	3.7 (3.3–4.4)	0.57
	PTA	-1.1 (-4.3–1.4)	-0.9 (-4.1–1.1)	-1.2 (-6.5–0.5)	0.81
	ROM	**2.6 (1.8–3.6)**	**3.8 (2.1–6)**	**5.8 (4.6–7.2)**	0.003^*^^,^^**b**^
**TRUNK lateral bending**	AVS	2.7 (1.8–4)	2.5 (1.4–3.9)	3.8 (3.4–4.7)	0.05
	PTA	-3.5 (-7.3–0.6)	-1.3 (-3.8–1.4)	-3.7 (-4.7–(-1.9))	0.10
	ROM	3.3 (2.8–5.1)	3.8 (2.1–5.6)	6 (5.3–6.8)	0.04
**TRUNK rotation**	AVS	2.1 (1.3–4.2)	3.6 (2.4–6.1)	5.3 (3.6–6.7)	0.07
	PTA	-0.8 (-3.1–1.8)	1.5 (-0.8–4.2)	-1.1 (-3.8–3.9)	0.23
	ROM	3.8 (2.4–6.2)	4.6 (3–7.6)	7.3 (5.8–8.4)	0.02

Me, median; IQR, interquartile ranges; MACS, Manual Ability Classification System; AVS, arm variable score; PTA, point of task achievement; ROM, range of motion

*, significant based on Holm-Bonferroni multiple level of sequentially rejective significance levels of three combinations; significant post-hoc differences

^a^, between MACS I and II

^b^, between MACS I and III

^c^, between II and III

^d^, no significant post-hoc differences.

**Table 3:** Statistical comparison of the joint specific parameters (Me, IQR) between MACS I, II and III during reach-to-grasp.

		MACS l (n = 15)	MACS ll (n = 26)	MACS lll (n = 9)	p-value
**WRIST flexion/extension**	AVS	**11.1 (7.7–13.4)**	**19.5 (11.3–31.4)**	**49.4 (31.1–57.2)**	<0.0001^*^^,^^**a**^^**,**^^**b**^^**,**^^**c**^
	PTA	**-7.3 (-12.3–1.7)**	**2.7 (-9.2–14.8)**	**8.7 (7.6–14.9)**	0.003^*^^,^^**b**^
	ROM	**25.8 (22.6–34.7)**	**27.8 (25.1–37.7)**	**50.9 (28.9–61)**	0.01^*^^,^^**b**^^**,**^^**c**^
**WRIST ulnar/radial deviation**	AVS	8.0 (6.2–19)	7.5 (5.3–17.7)	12.2 (5.6–17.9)	0.94
	PTA	-4.7 (-17.1–4.5)	3.8 (-2.5–11.1)	5.7 (-4.3–19.41	0.05
	ROM	15.3 (13.4–19.7)	14.0 (9.3–17.1)	14.8 (13.1–18)	0.44
**ELBOW pro/supination**	AVS	**13.6 (9.6–18.9)**	**28.3 (14.8–42.2)**	**43.2 (18–49.7)**	0.003^*^^,^^**a**^^**,**^^**b**^
	PTA	**101.1 (82.7–103.93**	**124.8 (106–141.3)**	**146.6 (113.9–156.8)**	<0.0001^*^^,^^**a**^^**,**^^**b**^
	ROM	**50.1 (43.6–56.5)**	**30.4 (24.2–41.5)**	**30.3 (25–33.7)**	<0.0001^*^^,^^**a**^^**,**^^**b**^
**ELBOW flexion/extension**	AVS	**14.8 (10–17.6)**	**25.3 (20.3–35.5)**	**28.7 (25.9–46)**	0.0001^*^^,^^**a**^^**,**^^**b**^
	PTA	**36.5 (25.7–45.8)**	**61.2 (52.9–70.9)**	**64.5 (64.3–77.9)**	<0.0001^*^^,^^**a**^^**,**^^**b**^
	ROM	**28.7 (19.6–36.2)**	**17.5 (12.9–25.2)**	**22.8 (18.6–35.5)**	0.03^*^^,^^**d**^
**SHOULDER elevation plane**	AVS	14.8 (11.3–18.7)	17.9 (12–27.6)	17.9 (15.1–22.9)	0.35
	PTA	77.3 (69.7–83.9)	69.3 (63.1–78.7)	69.7 (67.3–74.2)	0.22
	ROM	40.5 (34.8–50.8)	39.7 (28.7–50.3)	36.6 (28.4–40.6)	0.48
**SHOULDER elevation**	AVS	**8.1 (6.6–13.6)**	**11.9 (9.4–15.2)**	**16.5 (13.7–16.7)**	0.004^*^^,^^**b**^^**,**^^**c**^
	PTA	-78.7 (-84.5–(-66.6))	-72.1 (-78.5–(-67.0))	-64.7 (-79.4–(-60.7))	0.14
	ROM	53.9 (46.9–61.8)	50.4 (43.6–54.8)	44.4 (42.2–51.6)	0.06
**SHOULDER rotation**	AVS	14 (8.6–25.1)	15.3 (11.8–19.2)	19.8 (13.6–23.1)	0.73
	PTA	-52.5 (-63.3–(-45.9))	-69.4 (-71.2–(-58.5))	-70.1 (-82.5–(-61.9))	0.03
	ROM	37.5 (28.7–47.5)	39.4 (30.9–53)	32.7 (28.1–39)	0.64
**SCAPULA pro/retraction**	AVS	6.9 (4.4–11.1)	6.8 (4.4–9.8)	8.4 (5–10)	0.96
	PTA	50.9 (47.5–56.6)	48.2 (43.7–53.8)	49.8 (49.1–52.5)	0.47
	ROM	14.8 (12.9–19)	12.8 (9.2–16.1)	13.5 (11.2–15.8)	0.13
**SCAPULA medial/lateral rotation**	AVS	6.6 (4.2–12.4)	7.3 (4.3–13.4)	13.1 (6.8–16.8)	0.17
	PTA	-19.9 (-25.2–(-12.5))	-16.8 (-20–(-7.4))	-4.2 (-11.3–(-2))	0.02
	ROM	26.1 (20.2–28.6)	24.7 (19.5–27.6)	22.3 (18.2–24.7)	0.38
**SCAPULA anterior/posterior tilting**	AVS	6.2 (3.4–14.1)	5.6 (4.2–10.7)	5.5 (2.8–7.6)	0.30
	PTA	-1.0 (-13.2–3.6)	-1.5 (-7.8–4.4)	-2.5 (-6.2–1.7)	0.73
	ROM	10.9 (7.8–12.1)	10.3 (8.2–18.0)	9.8 (8.8–14.9)	0.84
**TRUNK flexion/extension**	AVS	4.1 (1.9–7.2)	4.5 (3.1–6.6)	6.8 (4.6–8.2)	0.26
	PTA	1.3 (-0.6–6.3)	6.4 (2.0–11.8)	3.4 (2.1–6.3)	0.17
	ROM	**3.5 (2.4–5.6)**	**6.4 (5.3–12.2)**	**10.1 (7.8–10.8)**	0.0006^*^^,^^**a**^^**,**^^**b**^
**TRUNK lateral bending**	AVS	**2.5 (1.8–4.2)**	**3.9 (2.9–6.8)**	**6.8 (3.2–9.4)**	0.01^*^^,^^**d**^
	PTA	**-7.9 (-11.4 -(-5.8)**	**-3.1 (-6.2–0.3)**	**-1.6 (-4.8–6.7)**	0.002^*^^,^^**a**^^**,**^^**b**^
	ROM	6.7 (5.1–10.1)	6.5 (4.5–9.1)	8.2 (7.1–8.5)	0.50
**TRUNK rotation**	AVS	**3.4 (2.5–5.7)**	**6.8 (4.9–10)**	**5.6 (4.6–8.3)**	0.01^*^^,^^**a**^
	PTA	12.7 (7.3–16.9)	16.4 (12.4–22)	15.4 (12.8–22.5)	0.11
	ROM	**10.7 (6.8–14.7)**	**14.1 (11.9–17.5)**	**15.8 (13.7–20.2)**	0.02^*^^,^^d^

Me, median; IQR, interquartile ranges; MACS, Manual Ability Classification System; AVS, arm variable score; PTA, point of task achievement; ROM, range of motion

*, significant based on Holm-Bonferroni multiple level of sequentially rejective significance levels of three combinations; significant post-hoc differences

^a^, between MACS I and II

^b^, between MACS I and III

^c^, between II and III

^d^, no significant post-hoc differences.

### Relation between sensorimotor impairments and kinematic parameters

#### Global parameters

For all three tasks, moderate to high correlations were found between the severity of motor impairments and the total amount of movement pathology, i.e. the APS (Table 4, r = 0.49 to -0.73). Sensory deficits showed only low correlations with the APS (r = -0.39 to -0.46). Lower grip force, lower muscle strength and higher muscle tone were also moderately correlated with longer movement durations (r = 0.47 to -0.66). For RGV, low to moderate correlations were found between more severe sensory and motor impairments and less straight hand trajectories (r = -0.39 to -0.68).

**Table 4:** Correlation coefficients between sensorimotor impairments and global kinematic parameters.

	Grip force^a^	Muscle strength^b^	Muscle tone^b^	TPD^b^	Stereognosis^b^
**HTH**					
APS (°)	-0.66****	-0.71****	0.52***	-0.40**	-0.46***
Duration (s)	-0.62****	-0.55****	0.47***	-	-
TimeVmax (%)	0.39**	0.39**	-0.30*	-	-
Vmax (m/s)	-	**-**	**-**	-	-
TS	-	-	-	-	-
**HTM**					
APS (°)	-0.63****	-0.65****	0.54****	-0.40**	-0.40**
Duration (s)	-0.66****	-0.52***	0.48***	-	-
TimeVmax (%)	0.51***	0.30*	-	-	-
Vmax (m/s)	-	0.34*	**-**	-	-
TS	-0.38**	-	-	-	-
**RGV**					
APS (°)	-0.69****	-0.73****	0.49***	-0.39**	-0.44**
Duration (s)	-0.63****	-0.56****	0.54****	-	-
TimeVmax (%)	0.47***	0.46**	-0.42**	-	-
Vmax (m/s)	-	-	-	-	-
TS	-0.54****	-0.68****	0.44**	-0.50***	-0.39**

^a^, pearson correlation coefficient

^b^, biserial correlation coefficient; TPD, two-point discrimination; TimeVmax: timing of maximal velocity; Vmax, maximal velocity; TS, trajectory straightness; HTH, hand-to-head; HTM, hand-to-mouth; RGV, reach-to-grasp vertically; only correlations above 0.30 are displayed

*, p<0.05

**, p<0.01

***, p<0.001

****, p<0.0001.

#### Joint specific parameters

For all three tasks, low to high correlations were found between the severity of sensorimotor impairments and movement deviations at the individual joints, i.e. reduced grip force, lower muscle strength or increased muscle tone were associated with increased wrist flexion (AVS, angle at PTA; r = 0.41 to -0.74) and elbow pronation (AVS, angle at PTA, ROM; r = 0.33 to -0.65) (Tables 5–7). These motor impairments also correlated with reduced elbow extension during RGV (Table 7; AVS, angle at PTA, ROM; r = 0.32 to -0.68) and with increased shoulder elevation (AVS, angle at PTA, ROM; r = -0.32 to 0.60) and increased scapular lateral rotation during HTM (Table 6; ROM; r = -0.36 to -0.62). A low correlation was further found between reduced grip force and muscle strength and increased shoulder external rotation at PTA during HTM and RGV (PTA; r = 0.35 and r = 0.35), but not during HTH (Table 5). At the level of the trunk, significant but mostly lower correlations were found with grip force and muscle strength during RGV (r = -0.34 to -0.53) and with grip force during HTH (r = -0.33 to -0.46). Finally, low correlations were found between sensory deficits and increased movement deviations of wrist flexion and elbow pronation for all three tasks (r = -0.34 to -0.49), as well as increased elbow flexion during RGV (r = -0.36 to -0.44). Only for RGV, low to moderate correlations were found between the severity of sensory deficits and the amount of trunk flexion/extension and lateral bending (r = -0.34 to -0.54).

**Table 5:** Correlation coefficients between sensorimotor impairments and joint specific parameters during hand-to-head.

		Grip force^a^	Muscle strength^b^	Muscle tone^b^	TPD^b^	Stereognosis^b^
**WRIST flexion/extension**	AVS	-0.56*	-0.63*	0.45*	-0.49*	-0.47*
	PTA	-0.43**	-0.42**	0.37**	-	-0.31
	ROM	-	-	-	-	-
**WRIST ulnar/radial deviation**	AVS	-	-	-	-	-
	PTA	-0.32**	-0.44*	-	-	-
	ROM	0.39*	0.38**	-0.37*	0.35*	-
**ELBOW pro/supination**	AVS	-0.46**	-0.55***	0.34**	-0.34*	-0.32
	PTA	-0.44***	-0.57**	0.33***	-	-
	ROM	0.49*	0.64*	-0.50*	-	0.30
**ELBOW flexion/extension**	AVS	-0.30	-0.31	-	-	-
	PTA	-	-	-	-	-
	ROM	-	-	-	-	-
**SHOULDER elevation plane**	AVS	-	-	-	-	-
	PTA	-	-	-	-	-
	ROM	-	-	-	-	-
**SHOULDER elevation**	AVS	-	-	-	-	-
	PTA	-	-	-	-	-
	ROM	-	-	-	-	-0.34
**SHOULDER rotation**	AVS	-	-	-	-	-
	PTA	-	-	-	-	-
	ROM	-	-	-	-	-
**SCAPULA pro/retraction**	AVS	-	-	-	-	-0.35*
	PTA	-0.38*	-	-	-	-0.33**
	ROM	-	-	-	-	-
**SCAPULA medial/lateral rotation**	AVS	-	-	-	-	-
	PTA	-	-	-	-	-
	ROM	-	-	-	-	-
**SCAPULA anterior/posterior tilting**	AVS	-	-	-	-	-
	PTA	-	-	-	-	-
	ROM	-	-	-	-	-
**TRUNK flexion/extension**	AVS	-0.33***	-	-	-	-
	PTA	0.33**	-	-	0.41*	-
	ROM	-0.46*	-0.36*	-	-0.40**	-
**TRUNK lateral bending**	AVS	-0.37*	-0.35*	-	-0.32	-
	PTA	-	-	-	-	-
	ROM	-0.33**	-	-	-	-
**TRUNK rotation**	AVS	-0.45*	-0.31	-	-	-
	PTA	-0.38**	-	-	-	-
	ROM	-0.31***	-	-	-	-

^a^, pearson correlation coefficient

^b^, biserial correlation coefficient; TPD, two-point discrimination; AVS, arm variable score; PTA, point of task achievement; ROM, range of motion; only correlations above 0.30 are displayed

*, p<0.0167

**, p<0.025

***, p<0.05; non-bold, not significant after Holm-Bonferroni method.

**Table 6:** Correlation coefficients between sensorimotor impairments and joint specific parameters during hand-to-mouth.

		Grip force^a^	Muscle strength^b^	Muscle tone^b^	TPD^b^	Stereognosis^b^
**WRIST flexion/extension**	AVS	-0.54*	-0.67*	0.45*	-0.44*	-0.35*
	PTA	-0.43**	-0.56**	0.41**	-0.31	-
	ROM	-0.34***	-	-	-	-0.31
**WRIST ulnar/radial deviation**	AVS	-	-	-	-	-
	PTA	-	-	-	-	-
	ROM	-	-	-	-	-
**ELBOW pro/supination**	AVS	-0.46***	-0.45**	0.36***	-0.39*	-0.43*
	PTA	-0.54**	-0.43***	0.44**	-0.37**	-0.40***
	ROM	0.57*	0.46*	-0.61*	0.37***	0.42**
**ELBOW flexion/extension**	AVS	-0.31	-	-	-	-
	PTA	-	-	-	-	-
	ROM	0.32	-	-0.42*	-	-
**SHOULDER elevation plane**	AVS	-	-	-	-	-
	PTA	-	0.32	-	-	-
	ROM	-	-	-	-	-
**SHOULDER elevation**	AVS	-0.32***	-0.42***	0.35***	-	-
	PTA	0.41*	0.60*	-0.50*	0.35*	0.32
	ROM	-0.41**	-0.59**	0.40**	-	-
**SHOULDER rotation**	AVS	-	-	-	-	-
	PTA	0.35*	-	-	-	-
	ROM	-	-	-	-	-
**SCAPULA pro/retraction**	AVS	-	-	-	-	-
	PTA	-	-	-	-	-
	ROM	-	-	-	-	-
**SCAPULA medial/lateral rotation**	AVS	-	-	-	-	-
	PTA	-	0.32	-	-	-
	ROM	-0.36*	-0.62*	**-**	-0.35*	-0.31
**SCAPULA anterior/posterior tilting**	AVS	-	-	-	-	-
	PTA	-	-	-	-	-
	ROM	-	-	-	-	-
**TRUNK flexion/extension**	AVS	-0.32**	-	-	-	-
	PTA	0.30***	-	-	-	-
	ROM	-0.55*	-0.47*	0.45*	-	-
**TRUNK lateral bending**	AVS	-	-	-	-0.31	-0.30
	PTA	-	-	-	-	-
	ROM	-	-0.37*	-	-	-
**TRUNK rotation**	AVS	-0.42**	-	0.32	-	-
	PTA	-	-	-	-	-
	ROM	-0.55*	-0.41*	0.39*	-	-

^a^, pearson correlation coefficient

^b^, biserial correlation coefficient; TPD, two-point discrimination; AVS, arm variable score; PTA, point of task achievement; ROM, range of motion; only correlations above 0.30 are displayed

*, p<0.0167

**, p<0.025

***, p<0.05; non-bold, not significant after Holm-Bonferroni method.

**Table 7:** Correlation coefficients between sensorimotor impairments and joint specific parameters during reach-to-grasp.

		Grip force^a^	Muscle strength^b^	Muscle tone^b^	TPD^b^	Stereognosis^b^
**WRIST flexion/extension**	AVS	-0.60*	-0.74*	0.44*	-0.40**	-0.45*
	PTA	-0.48**	-0.41***	0.38**	-	-
	ROM	-0.39***	-0.56**	-	-0.45*	-0.40**
**WRIST ulnar/radial** **deviation**	AVS	-	-	-	-	-
	PTA	-0.36*	-0.47*	-	-	-
	ROM	-	-	-	-	-
**ELBOW pro/supination**	AVS	-0.57**	-0.61**	0.39***	-0.32	-0.30
	PTA	-0.62*	-0.65*	0.44**	-0.35*	-0.31
	ROM	0.52***	0.56***	-0.55*	-	0.35*
**ELBOW flexion/extension**	AVS	-0.59**	-0.56**	0.37***	-0.30	-0.36**
	PTA	-0.68*	-0.60*	0.50*	-0.39*	-0.44*
	ROM	0.32***	-	-0.41**	-	-
**SHOULDER elevation plane**	AVS	-	-	-	-	-
	PTA	-	-	-	-	-
	ROM	-	-	-	-	-
**SHOULDER elevation**	AVS	-0.35*	-0.40*	-	-0.38*	-
	PTA	-	-	-	-	
	ROM	0.30	-	-0.34	-	0.30
**SHOULDER rotation**	AVS	-	-	-	-	-
	PTA	0.35*	0.37*	-	-	-
	ROM	-	-	-	-	-
**SCAPULA pro/retraction**	AVS	-	-	-	-	-
	PTA	-	-	-	-	-
	ROM	-	-	-0.30	-	-
**SCAPULA medial/lateral rotation**	AVS	-	-	-	-	-
	PTA	0.38*	-	-	-	-
	ROM	-	-	-	-	-
**SCAPULA anterior/posterior tilting**	AVS	-	-	-	-	-
	PTA	-	-	-	-	r = 0.30
	ROM	-	-	-	-	-
**TRUNK flexion/extension**	AVS	-	-	-	-0.54*	-0.43**
	PTA	-	-	-	-	-
	ROM	-0.53*	-0.52*	0.32	-0.48**	-0.45*
**TRUNK lateral bending**	AVS	-0.42**	-0.43*	0.34	-0.34**	-
	PTA	-0.48*	-0.40***	0.31	-0.39*	-0.32
	ROM	-	-0.42**	-	-	-
**TRUNK rotation**	AVS	-0.36***	-0.47**	0.41*	-	-
	PTA	-0.42**	-0.34***	-	-	-
	ROM	-0.53*	-0.52*	0.40**	-	-

^a^, pearson correlation coefficient

^b^, biserial correlation coefficient; TPD, two-point discrimination; AVS, arm variable score; PTA, point of task achievement; ROM, range of motion; only correlations above 0.30 are displayed

*, p<0.0167

**, p<0.025

***, p<0.05; non-bold, not significant after Holm-Bonferroni method.

### Relation of AHA and MA2 with kinematic parameters

#### Global parameters

For all three tasks, we found moderate to high correlations between lower levels of bimanual performance and unimanual capacity and more movement pathology (APS) and longer movement durations (Table 8, r = -0.50 to -0.87). Furthermore, lower scores on the AHA and MA2 correlated low to moderately with less straight hand trajectories during HTM (r = -0.36 to -0.47) and RGV (r = -0.64 to -0.71), respectively.

**Table 8:** Correlation coefficients between AHA and MA2 with global kinematic parameters.

	AHA^a^	MA2 ROM^a^	MA2 Acc^a^	MA2 Dex^a^	MA2 Fl^a^
**HTH**					
APS (°)	-0.68****	-0.87****	-0.78****	-0.74****	-0.71****
Duration (s)	-0.58****	-0.65****	-0.36*	-0.57****	-0.50***
TimeVmax (%)	0.41**	-0.39*	-	0.38*	-
Vmax (m/s)	-	**-**	-	-	-
TS	-	-	-	-	-
**HTM**					
APS (°)	-0.67****	-0.84****	-0.73****	-0.71****	-0.64****
Duration (s)	-0.61****	-0.58****	-0.33*	-0.63****	-0.55****
TimeVmax (%)	0.45**	0.42**	-	0.44**	0.43**
Vmax (m/s)	-	**-**	**-**	**-**	-
TS	-0.47**	-0.36*	-	-0.43**	-0.44**
**RGV**					
APS (°)	-0.75****	-0.84****	-0.70****	-0.72****	-0.69****
Duration (s)	-0.66****	-0.66****	-0.50***	-0.74****	-0.72****
TimeVmax (%)	0.59****	0.55***	0.42**	0.61****	0.61****
Vmax (m/s)	-	-	-	-	-
TS	-0.64****	-0.66****	-0.65****	-0.71****	-0.67****

^a^, pearson correlation coefficient; MA2, Melbourne Assessment 2; ROM, range of motion; Acc, accuracy; Dex, dexterity; Fl, fluency; AHA, Assisting Hand Assessment; TimeVmax: timing of maximal velocity; Vmax, maximal velocity; IC, index of curvature; HTH, hand-to-head; HTM, hand-to-mouth; RGV, reach-to-grasp vertically; only correlations above 0.30 are displayed

*, p<0.05

**, p<0.01

***, p<0.001

****, p<0.0001.

#### Joint specific parameters

For all three tasks, low to high correlations were found between lower levels of bimanual performance and unimanual capacity and higher AVS of wrist and elbow flexion/extension (r = -0.35 to -0.78), increased wrist flexion at PTA (r = -0.39 to -0.60) and more deficits of elbow pro/supination (AVS, angle at PTA, ROM; r = 0.30 to -0.76) (Tables 9–11). In addition, moderate to high correlations were found for RGV between lower scores on the AHA and MA2 and reduced elbow extension (r = -0.50 to -0.75). Proximally, only low to moderate correlations were found. Low correlations were shown between lower AHA and MA2 scores and more deficits of scapula pro/retraction during HTH (Table 9; AVS, angle at PTA; r = -0.35 to -0.48). For HTM (Table 10), lower scores on the AHA and MA2 were associated with more deficits in shoulder elevation (AVS, angle at PTA, ROM; r = -0.30 to -0.68) and scapula lateral rotation (ROM; r = -0.48 to -0.60). During RGV (Table 11), lower AHA and MA2 scores correlated with deviations in shoulder elevation and external rotation (AVS, angle at PTA; r = -0.39 to -0.44, r = 0.38 to 0.50). Finally, children with lower AHA and MA2 scores had a larger ROM of trunk flexion/extension (r = -0.37 to -0.66) and higher AVS for trunk rotation for all three tasks (r = -0.35 to -0.44), and more trunk lateral bending during RGV (AVS, angle at PTA, ROM; r = -0.31 to -0.54).

**Table 9:** Correlation coefficients between AHA and MA2 with joint specific parameters during hand-to-head.

		AHA^a^	MA2 ROM^a^	MA2 Acc^a^	MA2 Dex^a^	MA2 Fl^a^
**WRIST flexion/extension**	AVS	-0.69*	-0.74*	-0.64*	-0.69*	-0.56*
	PTA	-0.56**	-0.60**	-0.48**	-0.59**	-0.46**
	ROM	-	-	-	-	-
**WRIST ulnar/radial deviation**	AVS	-	-	-	-	-
	PTA	-0.38*	-0.39*	-0.30	-0.34	-0.32
	ROM	0.32	-	-	0.31	0.30
**ELBOW pro/supination**	AVS	-0.46**	-0.66*	-0.60*	-0.53**	-0.53*
	PTA	-0.41***	-0.60**	-0.50***	-048***	-0.48***
	ROM	0.52*	0.57***	0.53**	0.57*	0.52**
**ELBOW flexion/extension**	AVS	-0.34	-0.44*	-0.34	-0.44*	-0.36*
	PTA	-	-	-	-	-
	ROM	-	-	-	-	-
**SHOULDER elevation plane**	AVS	-	-	-	-	-
	PTA	-	-	-	-	-
	ROM	-	-	-	-	-
**SHOULDER elevation**	AVS	-	-	-	-	-
	PTA	-	-	-	-	-
	ROM	-	-	-	-	-
**SHOULDER rotation**	AVS	-	-	-	-	-
	PTA	0.30	0.32	-	-	-
	ROM	-	-	-	-	-
**SCAPULA pro/retraction**	AVS	-0.30	-0.35**	-0.36*	-0.44**	-
	PTA	-0.41*	-0.37*	-	-0.48*	-0.30
	ROM	-	-	-	-	-
**SCAPULA medial/lateral rotation**	AVS	-	-	-	-	-
	PTA	-	-	-	-	-
	ROM	-	-	-	-	-
**SCAPULA anterior/posterior tilting**	AVS	-	-	-	-	-
	PTA	-	-	-	-	-
	ROM	-	-	-	-	-
**TRUNK flexion/extension**	AVS	-	-	-	-	-0.41***
	PTA	-	-	-	-	0.48**
	ROM	-0.39*	-0.43*	-	-0.37*	-0.49*
**TRUNK lateral bending**	AVS	-	-	-0.32	-	-
	PTA	-	-	-	-	-
	ROM	-	-0.32	-	-	-
**TRUNK rotation**	AVS	-0.44*	-0.41*	-0.33	-0.38*	-0.44*
	PTA	-0.41**	-	-	-	-0.40**
	ROM	-	-	-	-	-

^a^, pearson correlation coefficient; MA2, Melbourne Assessment 2; ROM, range of motion; Acc, accuracy; Dex, dexterity; Fl, fluency; AHA, Assisting Hand Assessment; AVS, arm variable score; PTA, point of task achievement; only correlations above 0.30 are displayed

*, p<0.0167

**, p<0.025

***, p<0.05; non-bold, not significant after Holm-Bonferroni method.

**Table 10:** Correlation coefficients between AHA and MA2 with joint specific parameters during hand-to-mouth.

		AHA^a^	MA2 ROM^a^	MA2 Acc^a^	MA2 Dex^a^	MA2 Fl^a^
**WRIST flexion/extension**	AVS	-0.61*	-0.69*	-0.62*	-0.59*	0.56*
	PTA	-0.44**	-0.49**	-0.50**	-0.42**	-0.44**
	ROM	-0.37***	-0.33***	-	-	-
**WRIST ulnar/radial deviation**	AVS	-	-	-	-	-
	PTA	-	-	-	-	-
	ROM	-	-	-	-	-
**ELBOW pro/supination**	AVS	-0.49***	-0.58**	-0.49*	-0.56***	-0.48***
	PTA	-0.57*	-0.62*	-0.46**	-0.62*	-0.55**
	ROM	0.53**	0.50***	0.30***	0.60**	0.57*
**ELBOW flexion/extension**	AVS	-0.35*	-0.53*	-0.50*	-0.52*	-0.45*
	PTA	-	-0.33***	-	-	-0.31
	ROM	0.31	0.35**	-	0.33	-
**SHOULDER elevation plane**	AVS	-	-	-	-	-
	PTA	-	-	0.42*	-	0.32
	ROM	-	-	-	-	-
**SHOULDER elevation**	AVS	-0.30***	-0.46***	-0.39***	-0.49***	-0.31***
	PTA	0.54**	0.65**	0.53**	0.66*	0.51*
	ROM	-0.56*	-0.68*	-0.55*	-0.65**	-0.47**
**SHOULDER rotation**	AVS	-	-	-	-	-
	PTA	0.35	0.38*	-	0.32	-
	ROM	-	-	-	-	-
**SCAPULA pro/retraction**	AVS	-	-	-	-	-
	PTA	-	-	-	-	-
	ROM	-	-	-	-	-
**SCAPULA medial/lateral rotation**	AVS	-	-	-	-	-
	PTA	-	-	-	-	-
	ROM	-0.48*	-0.60*	-0.59*	-0.55*	-0.50*
**SCAPULA anterior/posterior tilting**	AVS	-	-	-	-	-
	PTA	-	-	-	-	-
	ROM	-	-	-	-	-
**TRUNK flexion/extension**	AVS	-	-	-	-	-0.36***
	PTA	-	-	-	-	0.36**
	ROM	-0.52*	-0.54*	-0.56*	-0.51*	-0.58*
**TRUNK lateral bending**	AVS	-	-	-0.40*	-	-
	PTA	-	-	0.36**	-	-
	ROM	-	-0.36*	-0.34***	-0.33	-
**TRUNK rotation**	AVS	-0.35**	-0.39**	-	-0.37**	-0.41**
	PTA	-	-	-	-	-
	ROM	-0.48*	-0.56*	-	-0.53*	-0.44*

^a^, pearson correlation coefficient; MA2, Melbourne Assessment 2; ROM, range of motion; Acc, accuracy; Dex, dexterity; Fl, fluency; AHA, Assisting Hand Assessment; AVS, arm variable score; PTA, point of task achievement; only correlations above 0.30 are displayed

*, p<110.0167

**, p<120.025

***, p<130.05; non-bold, not significant after Holm-Bonferroni method.

**Table 11:** Correlation coefficients between AHA and MA2 with joint specific parameters during reach-to-grasp.

		AHA^a^	MA2 ROM^a^	MA2 Acc^a^	MA2 Dex^a^	MA2 Fl^a^
**WRIST flexion/extension**	AVS	-0.74*	-0.78*	-0.66*	-0.71*	-0.64*
	PTA	-0.53**	-0.46***	-0.43***	-0.39***	-0.48**
	ROM	-0.47***	-0.57**	-0.46**	-0.62**	-0.43***
**WRIST ulnar/radial deviation**	AVS	-	-	-	-	-
	PTA	-0.43*	-0.53*	-0.34	-0.43*	-0.40*
	ROM	-	-	-	-	-
**ELBOW pro/supination**	AVS	-0.57***	-0.74**	-0.55**	-0.54***	-0.50**
	PTA	-0.64*	-0.76*	-0.57*	-0.62*	-0.59*
	ROM	0.61**	0.57***	0.47***	0.57**	0.48***
**ELBOW flexion/extension**	AVS	-0.62**	-0.65**	-0.45**	-0.60**	-0.55**
	PTA	-0.75*	-0.70*	-0.50*	-0.66*	-0.64*
	ROM	-	-	-	-	-
**SHOULDER elevation plane**	AVS	-0.30	-	-	-	-
	PTA	-	-	-	-	-
	ROM	-	-	-	-	-
**SHOULDER elevation**	AVS	-0.39*	-0.39*	-0.34	-0.40*	-0.44*
	PTA	-	-	-	-	-
	ROM	-	-	-	-	-
**SHOULDER rotation**	AVS	-	-	-	-	-
	PTA	0.39*	0.50*	0.38*	0.38*	0.35
	ROM	-	-	-	-	-
**SCAPULA pro/retraction**	AVS	-	-	-0.30	-	-
	PTA	-	-	-	-	-
	ROM	-	-	-	-	-
**SCAPULA medial/lateral rotation**	AVS	-	-	-	-	-
	PTA	0.39*	-0.35	-	-	0.38*
	ROM	-	-	-	-	-
**SCAPULA anterior/posterior tilting**	AVS	-	-	-	-	-
	PTA	-	-	-	-	-
	ROM	-	-	-	-	-
**TRUNK flexion/extension**	AVS	-0.30	-0.30	-0.51**	-	-0.36**
	PTA	-	-	-	-	-
	ROM	-0.64*	-0.57*	-0.66*	-0.55*	-0.63*
**TRUNK lateral bending**	AVS	-0.41**	-0.35***	-0.44**	-0.30	-0.35***
	PTA	-0.50*	-0.49*	-0.31***	-0.42*	-0.38**
	ROM	-0.31***	-0.38**	-0.54*	-0.33	-0.42*
**TRUNK rotation**	AVS	-0.41**	-0.37**	-0.33	-0.34	-0.38**
	PTA	-	-0.36***	-	-	-
	ROM	-0.45*	-0.52*	-0.39*	-0.43*	-0.49*

^a^, pearson correlation coefficient; MA2, Melbourne Assessment 2; ROM, range of motion; Acc, accuracy; Dex, dexterity; Fl, fluency; AHA, Assisting Hand Assessment; AVS, arm variable score; PTA, point of task achievement; only correlations above 0.30 are displayed

*, p<0.0167

**, p<0.025

***, p<0.05; non-bold, not significant after Holm-Bonferroni method.

### Regression analysis

Based on a forward stepwise regression analysis, only grip force was retained as a significant predictor of the variability in APS for the tasks HTH (R^2^ = 0.42; p<0.0001) and HTM (R^2^ = 0.38; p<0.0001). For RGV, 55% of the variability in APS was explained by a combination of grip force, age and MACS level. Grip force alone explained 44% of the variability in APS during RGV (R^2^ = 0.44; p = 0.002).

## Discussion

In this study, we assessed UL clinical and kinematic parameters in a large cohort of children with uCP with varying levels of manual abilities to attain a better understanding of the intricate relationship between sensorimotor impairments, activity measures and the specific kinematic deviations. Such insights are crucial to set individualized therapy goals and thus optimize the child’s UL functional potential. We found significant differences in UL movement pathology between children with different manual ability levels and demonstrated the adverse impact of muscle weakness, muscle tone and sensory deficits on UL kinematics, as well as the negative relation between aberrant UL kinematics and bimanual performance and unimanual capacity.

Thus far, only Klotz et al. reported differences in UL kinematics during six daily tasks between children with uCP with different MACS levels, i.e. children with MACS I moved quicker and used more elbow supination compared to MACS III and showed less trunk movement compared to MACS II and III [6]. Whilst their results correspond to the current study, Klotz et al. failed to demonstrate further significant differences, probably explained by the small sample size. Furthermore, these authors did not report wrist or scapula kinematics. We additionally showed that the more deviant UL kinematics in higher MACS levels were mostly characterized by increased wrist and elbow flexion and pronation, along with more shoulder elevation deviations and increased trunk flexion. The reported differences between children with different MACS levels exceed the previously reported standard error of measurements by Jaspers et al. [16], which further supports their clinical relevance. However, only standard error of measurements of duration, velocity and angles at PTA have been previously reported. For the remaining kinematic parameters, we found that all differences between MACS levels were larger than 10% of the mean, except for the difference in trajectory straightness during RGV between MACS I and II. Overall, we can assume that the reported differences in this study are large enough to represent true differences between children with different MACS levels. Assessing UL kinematics during various tasks, i.e. HTH, HTM and RGV, also demonstrated that differences between children with different MACS levels were most evident during RGV. This task requires the execution of elbow extension combined with supination which is particularly challenging for children with uCP, due to the impairing role of the biceps brachii muscle. Over-activity of this muscle impairs elbow extension, and simultaneously enforces elbow supination to assist the weakened supinator muscles in overcoming pronation forces [21,22]. The resulting limited ROM of elbow supination and extension further prevents proper placing of the hand around the cylinder leading to proximal compensations [21,22].

Further analyses showed that more severe sensorimotor impairments were significantly associated with higher APS, which again were most pronounced for RGV. These findings correspond to those of Jaspers et al. [12]), which is the only study that previously investigated the relation between the APS and motor impairments in children with uCP. Our study further added that more severe muscle weakness and muscle tone were mainly correlated with distal UL movement pathology, which was most evident for wrist flexion/extension and elbow pro/supination. This is not completely unexpected as muscle weakness and tone have been shown to be more pronounced at the wrist and elbow compared to the shoulder [23]. On the other hand, increased muscle weakness was also related to more deviant movement pathology of the shoulder, scapula and trunk. Lastly, grip force was the only predictor of the variability in total amount of movement pathology (APS) for HTH and HTM and the largest predictor for RGV. Together, these findings point towards the potential relevance of muscle strengthening as a treatment goal. On the other hand, reduced motor selectivity might also impact on UL movement pathology in children with uCP. The importance of selective motor control for gait performance was recently reported [24], though this area remains unexplored for the UL. Future studies incorporating the assessment of selective motor control, as measured with e.g. the ‘Selective Control of the Upper Extremity Scale’ [25], will increase our understanding of the role of motor selectivity in UL movement pathology.

Interestingly, more severe sensory deficits were also correlated with more deviant UL kinematics, especially at the level of the wrist, elbow and trunk. The impact of sensory deficits on UL movement pathology has not yet been reported and underlines the importance of intact sensory functions for normal movement and motor planning [26].

Finally, we explored the relation between deviant UL kinematics and bimanual performance and unimanual capacity, assessed with the AHA and MA2, respectively. Thus far, only Klotz et al. [6] investigated the relation between the ABILHAND-kids questionnaire, a measure of UL function in daily life, and UL kinematics. They reported moderate to high correlations between lower ABILHAND-kids scores and longer movement durations. We found similar correlations between movement duration and the AHA and MA2, and additionally demonstrated high correlations between lower AHA and MA2 scores and more severe movement pathology. Further inspection of the joint specific parameters showed most pronounced correlations between bimanual performance and unimanual capacity and wrist flexion/extension and elbow pro/supination movement deviations, which emphasizes the importance of these movements during the execution of functional tasks. Fewer correlations were found with shoulder, scapula and trunk kinematics. This finding suggests that conventional clinical scales might mainly capture distal motor function, i.e. wrist and elbow deficits, whereas 3DMA additionally provides details on proximal motor deficits, i.e. shoulder, scapula and trunk.

In conclusion, this study demonstrated the merit of a quantitative output as obtained with 3DMA, i.e. the assessment of movement deviations at the single joint level. In contrast, clinical scales provide mostly qualitative information and only a general description of UL dysfunction. As a result, 3DMA is particularly suited to use as an outcome measure to evaluate the efficacy of joint targeted interventions such as UL botulinum toxin-A injections or surgery. Furthermore, the moderate to high correlations between UL movement pathology and the AHA and MA2 scores highlight the adverse impact of deviant movement patterns on bimanual performance and unimanual capacity, mostly caused by wrist flexion and elbow pronation movement deviations. Hence, addressing these movement deviations via joint targeted interventions, might also improve unimanual capacity and bimanual performance [27]. The impact of distal motor deficits on proximal function in uCP [7,28] additionally stresses the importance of an assessment of the multiple degrees of freedom of all joints of the UL chain. For example, Fitoussi et al. [7] and Kreulen et al. [28] reported a decrease in shoulder and trunk compensatory movements based on a 3DMA following botulinum toxin-A injections in the forearm/hand [7] or following surgical correction of the elbow pronation deficit [7,28] in children with uCP. In case these intervention studies used only clinical scales, this information would have been lost. Similarly, it might be interesting to evaluate how therapeutic interventions such as CIMT or HABIT affect distal as well as proximal UL movement pathology. Both CIMT and HABIT are effective treatment modalities that aim to improve distal UL function [29]. However, the impact of these interventions on UL movement pathology remains unexplored. Lastly, it might be interesting to explore whether incorporating specific scapulothoracic training into the UL rehabilitation in children with uCP would further enhance their movement patterns. Hence, integrating 3DMA as an outcome tool in future studies assessing the effect of different therapy programs will further increase our understanding of its efficacy at the level of both the distal and proximal joints of the UL.

Some critical reflections are also warranted. First, in eight children the AHA, MA2 and the 3DMA were assessed at different points in time (<8 months). However, Klingels et al. [20] reported no significant time effects for these tests for a period of up to one year. Furthermore, apart from their standard physiotherapy, these children did not receive any additional treatments within that time gap. Secondly, we only included children with uCP with at least a minimal ability to actively grasp an object. Hence, current study results cannot be generalized to the more impaired children. However, children with very poor motor function usually have different therapy goals for which other assessments than an UL 3DMA might be more appropriate. Thirdly, the use of composite scores for muscle tone and strength might be criticized. However, movement deviations of one joint will inevitably influence other joints of the UL [7,28]. Moreover, mono and bi-articular muscles also play a differential role during multi-joint movements [30,31], i.e. mono-articular muscles mainly contribute to joint torque during shortening, whereas bi-articular muscles are activated to control the direction of the external force. Hence, scores at the single joint level might not fully capture how motor deficits affect UL movement pathology. Furthermore, using the same composite score across all kinematic variables reduces the number of dependent variables and thus the complexity introduced by multiple comparisons, and also facilitates the interpretation of current study results. Next, the use of the Modified Ashworth Scale has been previously debated due to low levels of reliability and validity [32]. Still, Klingels et al. [14] reported moderately high to very high levels of reliability for the composite scores of the Modified Ashworth Scale as well as for manual muscle strength testing for the UL in children with uCP. While instrumented measures for spasticity may provide a reliable and valid alternative to clinical testing as demonstrated by Bar-On et al. [33], its applicability for the muscle groups of the UL currently remains unexplored. Finally, children with botulinum toxin-A injections were included in case these injections occurred more than 6 months prior testing. However, virtually nothing is known on whether (repeated) botulinum toxin injections permanently change the UL movement pattern.

Nevertheless, it is important to note that the clinical application of UL 3DMA remains challenging, mainly because of the large variety of UL functions. This has caused a vast heterogeneity between existing studies regarding the employed protocol [3–10,21,28]. Here, we clearly showed that RGV discriminated best between children with different levels of manual ability and that correlations with the different clinical outcomes were stronger for RGV compared to the other two tasks. Therefore, we propose to incorporate this reach-to-grasp task in future studies in order to facilitate result comparison. Finally, studies thus far mostly focused on start or end angles of the movement [4,5], total active ROM [4–8,21,28] or indices of the severity of movement pathology [11,12]. Consequently, potential relevant information pertaining to the waveform itself might be lost, i.e. these variables provide no information at which point during the movement cycle the pathology is most pronounced. Recently, statistical parametric mapping (SPM) has been introduced to the field of biomechanics as a promising tool to overcome this issue [34]. The added value of SPM has already been proven in gait analysis [35] and may aid in the further detailed analyses of UL movement patterns in children with uCP.

## Conclusion

We found increased UL movement pathology in children with poorer manual abilities and demonstrated the adverse impact of muscle weakness, muscle tone and sensory deficits on UL kinematics, especially at the level of the wrist and elbow. Moreover, aberrant UL kinematics were associated with poor bimanual performance and unimanual capacity. Results further highlighted the importance of muscle strengthening as a treatment modality to decrease UL movement pathology as supported by the stronger correlations between muscle weakness and UL kinematics. Finally, the overall low to moderate correlations between joint kinematics and the different clinical measures suggest that a 3DMA provides added information regarding UL motor function, particularly for the proximal joints. Hence, integrating both methods seems clinically meaningful to obtain a comprehensive representation of all aspects of a child’s UL functioning.

## Supporting information

S1 Table Descriptive statistics of clinical outcomes according to MACS levels. *, Kruskal Wallis; ^¥^, Fisher’s exact test; MACS, Manual Ability Classification System; N, number; TPD, two-point discrimination; AHA, Assisting Hand Assessment; MA2, Melbourne Assessment 2; ROM, range of motion; N, number; Me, median; IQR, interquartile range; significant differences after post-hoc analyses ^a^, between MACS I and II; ^b^, between MACS I and III; ^c^, between II and III.
(DOCX)

S1 Fig Kinematic waveforms of all three MACS groups during HTH. Movement patterns of wrist, elbow, shoulder, scapula and trunk angles of children in MACS I (blue), MACS II (orange) and MACS III (yellow). The grey line indicates the average movement patterns of 60 typically developing children (shaded bar represents 1 standard deviation). Abbreviations: TDC, typically developing children; MACS, Manual Ability Classification System; HTH, hand-to-head.
(TIFF)

S2 Fig Kinematic waveforms of all three MACS groups during HTM. Movement patterns of wrist, elbow, shoulder, scapula and trunk angles of children in MACS I (blue), MACS II (orange) and MACS III (yellow). The grey line indicates the average movement patterns of 60 typically developing children (shaded bar represents 1 standard deviation). Abbreviations: TDC, typically developing children; MACS, Manual Ability Classification System; HTM, hand-to-mouth.
(TIFF)

S3 Fig Kinematic waveforms of all three MACS groups during RGV. Movement patterns of wrist, elbow, shoulder, scapula and trunk angles of children in MACS I (blue), MACS II (orange) and MACS III (yellow). The grey line indicates the average movement patterns of 60 typically developing children (shaded bar represents 1 standard deviation). Abbreviations: TDC, typically developing children; MACS, Manual Ability Classification System; RGV, reach-to-grasp vertically.
(TIFF)
