# Supplementary material for: Clinical assessment and three-dimensional movement analysis: An integrated approach for upper limb evaluation in children with unilateral cerebral palsy
Source: PLoS One. 2017 Jul 3;12(7):e0180196. doi: 10.1371/journal.pone.0180196 (PMC5495347; doi:10.1371/journal.pone.0180196)
Supplement: S1 Table — *, Kruskal Wallis; ¥, Fisher’s exact test; MACS, Manual Ability Classification System; N, number; TPD, two-point discrimination; AHA, Assisting Hand Assessment; MA2, Melbourne Assessment 2; ROM, range of motion; N, number; Me, median; IQR, interquartile range; significant differences after post-hoc analyses a, between MACS I and II; b, between MACS I and III; c, between II and III. (DOCX) [file pone.0180196.s001.docx]

| **S1 Table. Descriptive statistics of clinical outcomes according to MACS levels.** | | | | |  |
| --- | --- | --- | --- | --- | --- |
|  |  | **MACS I (N=15)** | **MACS II (N=26)** | **MACS III (N=9)** | **p-value** |
| **Grip force (%)^*^** | Me (IQR) | 78.6 (56.1 – 85.0) | 37.4 (28.2 - 51.6) | 28.6 (18.8 - 31.9) | <0.0001^a,b,c^ |
| **Muscle strength^*^** | Me (IQR) | 36 (35.5 - 37) | 33 (31.5 - 34) | 31.75 (30 - 32.5) | <0.0001^a,b^ |
| **Muscle tone^*^** | Me (IQR) | 3 (0 - 5.5) | 8 (6.5 - 10) | 8 (6.50 - 9.75) | <0.0001^a,b^ |
|  |  |  |  |  |  |
| **TPD^¥^** |  |  |  |  | 0.01^b^ |
| **Normal** | N (%) | 14 (93.3%) | 16 (61.5%) | 2 (25%) |  |
| **Impaired** | N (%) | 0 (0%) | 4 (15.4%) | 2 (25%) |  |
| **Absent** | N (%) | 1 (6.7%) | 5 (19.2%) | 5 (50%) |  |
| **Stereognosis^¥^** |  |  |  |  | 0.002^b,c^ |
| **Normal** | N (%) | 13 (86.7%) | 16 (61.5%) | 1 (11.1%) |  |
| **Impaired** | N (%) | 2 (13.3%) | 5 (19.2%) | 3 (33.3%) |  |
| **Absent** | N (%) | 0 (0%) | 5 (19.2%) | 5 (55.6%) |  |
|  |  |  |  |  |  |
| **AHA^*^** | Me (IQR) | 83 (77-86) | 61.5 (57 - 66) | 54 (52 - 55) | <0.0001^a,b,c^ |
| **MA2_ROM^*^** | Me (IQR) | 88.9 (81.8 - 92.6) | 68.5 (59.3 - 81.5) | 44.4 (44.4 - 55.6) | <0.0001^a,b,c^ |
| **MA2_Acc^*^** | Me (IQR) | 100 (96 - 100) | 96 (88 - 100) | 84 (80 - 86.4) | 0.0002^b,c^ |
| **MA2_Dext^*^** | Me (IQR) | 93.8 (87.5 - 100) | 68.8 (62.5 - 81.3) | 56.3 (43.8 - 56.3) | <0.0001^a,b,c^ |
| **MA_Fl^*^** | Me (IQR) | 95.2 (85.7 - 95.2) | 71.4 (61.9 - 81) | 61.9 (52.4 - 61.9) | <0.0001^a,b,c^ |
| ^*^, Kruskal Wallis; ^¥^, Fisher’s exact test; MACS, Manual Ability Classification System; N, number; TPD, two-point discrimination; AHA, Assisting Hand Assessment; MA2, Melbourne Assessment 2; ROM, range of motion; Acc, Accuracy; Dext, Dexterity; Fl, Fluency; N, number; Me, median; IQR, interquartile range; significant differences after post-hoc analyses ^a^, between MACS I and II; ^b^, between MACS I and III; ^c^, between II and III | | | | | |
