# Supplementary figures and images for: Clinical assessment and three-dimensional movement analysis: An integrated approach for upper limb evaluation in children with unilateral cerebral palsy
Source: PLoS One. 2017 Jul 3;12(7):e0180196. doi: 10.1371/journal.pone.0180196 (PMC5495347; doi:10.1371/journal.pone.0180196)

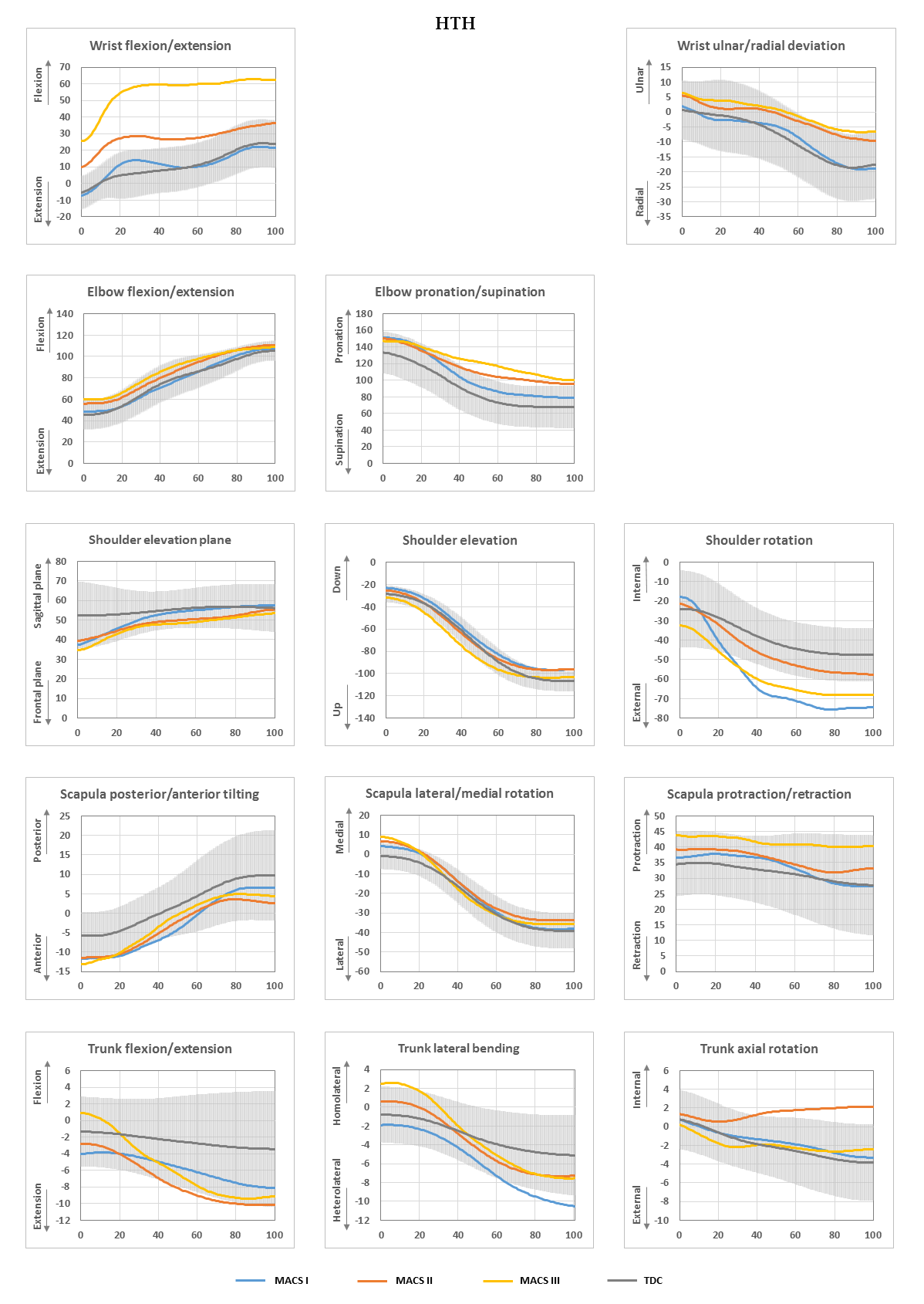

Supplement: S1 Fig — Movement patterns of wrist, elbow, shoulder, scapula and trunk angles of children in MACS I (blue), MACS II (orange) and MACS III (yellow). The grey line indicates the average movement patterns of 60 typically developing children (shaded bar represents 1 standard deviation). Abbreviations: TDC, typically developing children; MACS, Manual Ability Classification System; HTH, hand-to-head. (TIFF) [file pone.0180196.s002.tiff]

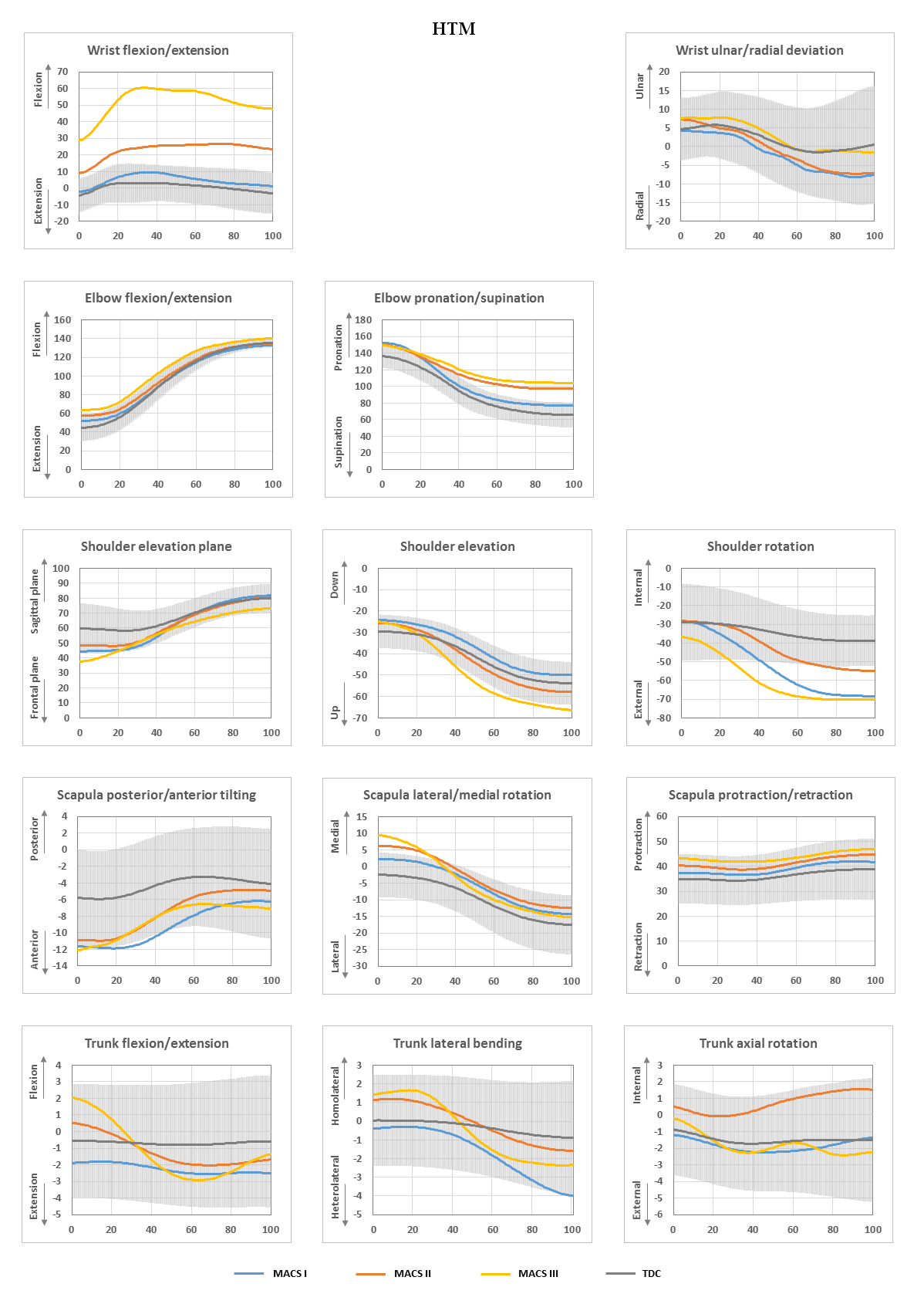

Supplement: S2 Fig — Movement patterns of wrist, elbow, shoulder, scapula and trunk angles of children in MACS I (blue), MACS II (orange) and MACS III (yellow). The grey line indicates the average movement patterns of 60 typically developing children (shaded bar represents 1 standard deviation). Abbreviations: TDC, typically developing children; MACS, Manual Ability Classification System; HTM, hand-to-mouth. (TIFF) [file pone.0180196.s003.tiff]

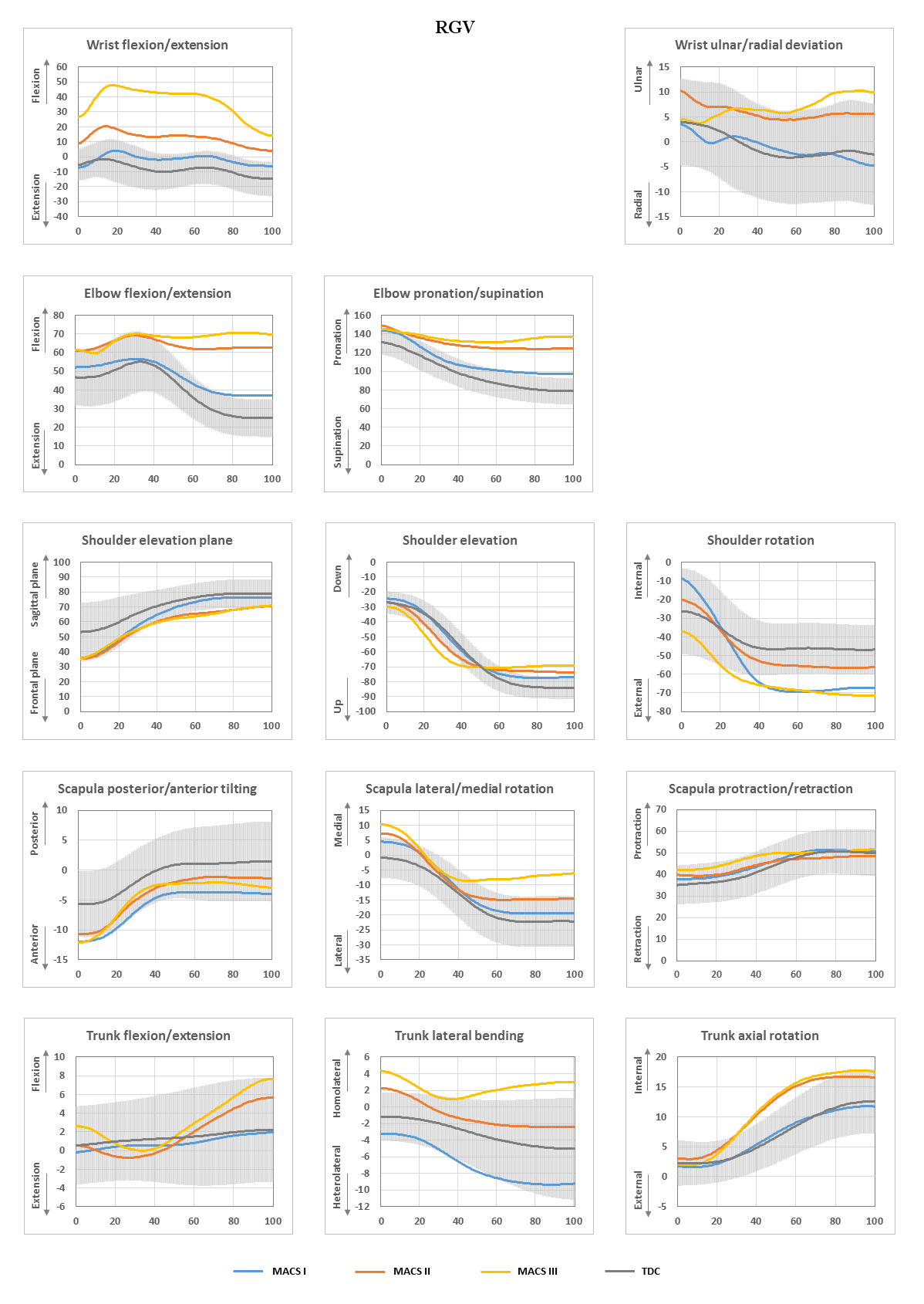

Supplement: S3 Fig — Movement patterns of wrist, elbow, shoulder, scapula and trunk angles of children in MACS I (blue), MACS II (orange) and MACS III (yellow). The grey line indicates the average movement patterns of 60 typically developing children (shaded bar represents 1 standard deviation). Abbreviations: TDC, typically developing children; MACS, Manual Ability Classification System; RGV, reach-to-grasp vertically. (TIFF) [file pone.0180196.s004.tiff]
